# SPOt: A novel and streamlined microarray platform for observing cellular tRNA levels

**DOI:** 10.1371/journal.pone.0177939

**Published:** 2017-05-17

**Authors:** Simon Grelet, Ariel McShane, Eveline Hok, Jensen Tomberlin, Philip H. Howe, Renaud Geslain

**Affiliations:** 1 Department of Biochemistry and Molecular Biology, MUSC, Charleston, SC, United States of America; 2 Laboratory of tRNA Biology, Department of Biology, College of Charleston, Charleston, SC, United States of America; Defense Threat Reduction Agency, UNITED STATES

## Abstract

Recent studies have placed transfer RNA (tRNA), a housekeeping molecule, in the heart of fundamental cellular processes such as embryonic development and tumor progression. Such discoveries were contingent on the concomitant development of methods able to deliver high-quality tRNA profiles. The present study describes the proof of concept obtained in *Escherichia coli* (*E*. *coli*) for an original tRNA analysis platform named SPOt (Streamlined Platform for Observing tRNA). This approach comprises three steps. First, *E*. *coli* cultures are spiked with radioactive orthophosphate; second, labeled total RNAs are trizol-extracted; third, RNA samples are hybridized on in-house printed microarrays and spot signals, the proxy for tRNA levels, are quantified by phosphorimaging. Features such as reproducibility and specificity were assessed using several tRNA subpopulations. Dynamic range and sensitivity were evaluated by overexpressing specific tRNA species. SPOt does not require any amplification or post-extraction labeling and can be adapted to any organism. It is modular and easily streamlined with popular techniques such as polysome fractionation to profile tRNAs interacting with ribosomes and actively engaged in translation. The biological relevance of these data is discussed in regards to codon usage, tRNA gene copy number, and position on the genome.

## Introduction

The genome of *Escherichia coli* supports the transcription of four different types of RNA: ribosomal RNA (rRNA), transfer RNA (tRNA), messenger RNA and small non-coding RNA (sRNA). rRNA and tRNA represent by far the most abundant and resilient RNA species. The 5S rRNA (120 nucleotides) and the 23S rRNA (2,904 nucleotides) are essential components of the large ribosomal subunit whereas the 16S rRNA (1,542 nucleotides) is part of the small subunit. Eighty-six intron-less tRNA genes are transcribed into 48 unique tRNA sequences organized in 40 different tRNA isoacceptor families (tRNAs with different anticodons) [1]. These tRNAs are transcribed from one to six gene copies all encoding the characteristic CCA 3’ -end. tRNA genes are organized as independent transcriptional entities or clustered in operons under the control of a single promoter. The intracellular level of a tRNA species at any given stage of the cellular cycle derives from a common multistep-process that starts with transcription, yielding a fully matured molecule that can be charged with an amino acid and incorporated into the translation machinery.

tRNA transcription promoters consist of two RNA polymerase recognition sequences: the −10 element also known as the Pribnow box and the −35 element. Elements beyond the promoter region have been identified; the function of these additional regulatory sequences remains elusive [2]. In addition to transcription, the production of functional tRNAs includes the trimming of 5’ and 3’ -ends and the addition of posttranscriptional modifications. Processing of 5’ leader sequences is catalyzed by the quasi-universal ribozyme RNase P while processing of 3’ trailers is initiated by endonucleolytic cleavage downstream of the terminal -CCA followed by recurring exonucleolytic trimming [3]. In polycistronic transcripts, endonucleolytic cleavage between individual tRNA sequences generates intermediates similar to monocistronic tRNA transcripts [4]. *E*. *coli* tRNA each harbor on average seven to eight posttranscriptional modifications supporting a range of cellular functions [5]. Modifications in the anticodon, in particular, modulate codon recognition and control frame shifting [6]. tRNAs that are imperfectly processed are polyadenylated and hydrolyzed by the combined action of RNase R and polynucleotidyl phosphorylase (PNPase) [7]. Matured *E*. *coli* tRNA can be organized in two classes according to their size. Class I tRNAs are the most abundant, their size range from 74 to 77 nucleotides. Class II tRNAs are 85- to 93-nucleotides long and include all Leucine, Serine, Selenocysteine and Tyrosine accepting species.

Until recently, tRNAs have been considered as housekeeping molecules with little to no regulatory functions. However, a growing body of evidence indicates that differential tRNA expression deeply influences the whole dynamic of translation, favoring or repressing the expression of particular proteins [8–10]. The collection of tRNA profiles represents a true technical challenge; because of their abundant post-transcriptional modifications and their stable secondary structures, tRNA levels cannot be directly or accurately measured using standard high-throughput sequencing or RT-PCR techniques [11]. Tailored and often ingenious approaches had to be designed to specifically assess cellular tRNA contents. Some of these methods include: (1) separation of metabolically labeled tRNAs by two-dimensional gel electrophoresis followed by systematic spot identification via Northern blot [12]; (2) identification of transcriptionally active tRNA genes by RNA polymerase III and chromatin immunoprecipitation followed by high-throughput sequencing (Chip-seq) [13]; (3) systematic quantification by Northern blot after standardization of probe activity [14]; (4) microarray analysis of tRNA samples labeled *in vitro* with fluorochromes or radioisotopes [15, 16]; (5) enzyme-assisted tRNA demodification followed by reverse transcription and high-throughput sequencing [17]; (6) hybridization of tRNA-specific DNA probes followed by quantitative PCR [8].

The study described in this paper contributes to the general and constant effort aimed at improving the quality of tRNA profiles and streamlining data collection. SPOt, Streamlined Platform for Observing tRNA is a novel, direct, and cost-effective method for rapid and accurate measurement of tRNA abundance in biological samples. For convenience, proof of concept was established in *E*. *coli*, due to its limited set of tRNA genes and a fast division rate. SPOt capitalizes on existing techniques such as *in vivo* tRNA body labeling and tRNA microarrays, which were combined here for the first time. This approach comprises three steps. First, *E*. *coli* cultures are spiked with radioactive orthophosphate; second, labeled total RNAs are trizol-extracted at mid-log phase; third, RNA samples are hybridized on microarrays printed in-house, and spot signals are quantified by phosphorimaging. SPOt does not require amplification or post-extraction labeling which are potential sources of bias. Features such as reproducibility, sensitivity, and specificity were thoroughly tested and were found to match or even surpass current standards for tRNA profiling.

## Methods

### Strain and growth media

The bacterial strain used in this study is HB101 a derivative of *E*. *coli* K12. It was grown in Luria Broth (LB) containing 10 g/l of tryptone, 5 g/l of yeast extract and 10 g/l of NaCl. The culture media was supplemented with 1 to 20 μCi/ml of [^32^P] Na_2_HPO_4_ (Perkin Elmer, NEX011001MC), 0.1 μM to 1 mM of IPTG and 5 μg/ml of ampicillin depending on the experiment performed. Cultures were initiated from starters grown overnight and diluted 100-fold into either 500 μl in 1.5 ml micro-centrifuge tubes (after poking the cap with a large diameter needle to allow proper aeration) or 250 μl in 96-well micro-plates. Cells were grown aerobically and under strong agitation in an Eppendorf Thermomixer or a shaker-incubator micro-plate reader (Accuskan FC, Fisher Scientific) to midlog phase (OD_600nm_ = 0.5) prior to sample collection by centrifugation. Competent cells were prepared by washing bacteria twice in ice cold 50 mM CaCl_2_, pH 6.1.

### tRNA overexpression

pTrc99 plasmids conferring ampicillin resistance and encoding *E*. *coli* Lys (TTT), Thr (CGT), Cys (GCA) or Leu (CAG) tRNA genes under the control of IPTG-inducible promoter were obtained from Dr. Gilbert Eriani (CNRS, France). Competent HB101 cells were heat shock transformed with 1 ng of plasmids from a maxiprep. Transformants were selected on media with ampicillin, grown and induced as described above.

### Polysome fractionation

*E*. *coli* cells were grown to midlog phase in 10 ml of LB supplemented with 10 μCi/ml of [^32^P] Na_2_HPO_4_ before being rapidly cooled to preserve the integrity of polysomes. All subsequent steps were performed at 4°C or indicated otherwise. Cells were centrifuged three minutes at 12,000xg, lysed ten minutes in 10 mM Tris-HCl, pH 8.0, 10 mM MgCl_2_ and 1mg.mL^-1^ Lysozyme and finally flash-frozen in a liquid nitrogen bath. The bacterial extract was then thawed on ice, supplemented with 30 μL of 10% sodium deoxycholate per ml of cell lysate and centrifuged at 16,000xg to pellet cell debris. The supernatant was loaded onto a linear 10–40% sucrose gradient (20mM Tris-HCl, pH 7.8, 10mM MgCl_2_, 100mM NH_4_Cl, 2mM DTT, 10 to 40% sucrose poured in 4 layers and incubated overnight for linear homogenization) and centrifuged three hours at 35,000 rpm using a Beckman SW-40ti rotor. Fractions were analyzed and collected using an ISCO UA6 in-line absorbance detector.

### Preparation of total RNA

Total RNAs were Trizol extracted from bacterial pellets or cell lysate fractioned on sucrose gradients following the manufacturer’s protocol (Ambion). RNA extracts were hybridized on microarrays (see below) or separated on 10% denaturing PAGE containing 7 M urea, stained twenty minutes in toluidine blue 0.05 mg/ml, imaged on a Biorad GelDoc and quantified with an Image Lab software.

### Array printing, hybridization and analysis

Standard tRNA microarray experiments consist of three steps starting from printing, followed by hybridization and finally data analysis. tRNA microarrays were manually printed with 42 70-mer DNA oligonucleotides complementary to the 3’ end of *E*. *coli* tRNA (terminal CCA excluded) using an eight-pin arrayer. The sequences of the DNA oligonucleotide probes as well as the probe layout on the arrays are provided as S1 File. Probes were spotted in eight replicates at 50 μM and cross-linked to amine coated glass slides by UV radiation (Spectroline, UV crosslinker). Arrays were blocked overnight in blocking solution (BlockIt, Arrayit Corporation), rinsed in water and dried by centrifugation. Prior to hybridization, slides were boiled in water for two minutes and dried. Pellets of radiolabeled RNAs obtained by Trizol extraction were dissolved in 180 μl of 2 X SSC, 0.1% (w/v) SDS and loaded onto arrays inside dedicated hybridization cassettes. The subsequent steps were performed on an automated hybridization-washing station (GeneTAC hybridization station) for maximum reproducibility. Hybridization was performed as following: 75°C (two minutes, gasket conditioning), 60°C (probe introduction), 90°C (five minutes, sample denaturation) and 60°C (4 hours, hybridization). Slides were then washed at 50°C twice with 2 X SSC, 0.1% (w/v) SDS, at 42°C twice with 0.1 X SSC, 0.1% SDS, and at 42°C twice with 0.1 X SSC. For a comprehensive protocol and description of required instruments, please refer to Swartz and Pan, 2016. Slides were then dried by centrifugation before being exposed onto a storage phosphor screen (BAS-IP SR 0813, Fujifilm) for 10 to 90 hours depending on signal strength and scanned at 50 μm resolution using a Typhoon FLA7000 phosphorimager. Radioactivity intensities at each probe spot were quantified and background-subtracted using the Image J software upgraded with a microarray profiler. Further data manipulation was performed with Microsoft Excel. Heat maps were generated using the Excel conditional formatting tool.

## Results and discussion

### Metabolic labeling of abundant cellular RNAs

Metabolic labeling refers to methods in which the cellular machinery of living cells is co-opted to steadily incorporate traceable agents into biomolecules. Many nucleotide analogs commonly used to label nucleic acids *in vivo* are antimetabolites that are detected by click- or immuno-reactions. Because of their relative toxicity such analogs are spiked in culture media for a short period of time near the experimental endpoint. Alternatively, controlled amounts of radioactive elements such as radioactive isotopes of phosphorus can be used for prolonged labeling of cofactors, metabolites, lipids, proteins, DNA and RNA without impact on cell viability [18, 19].

**Fig 1:**
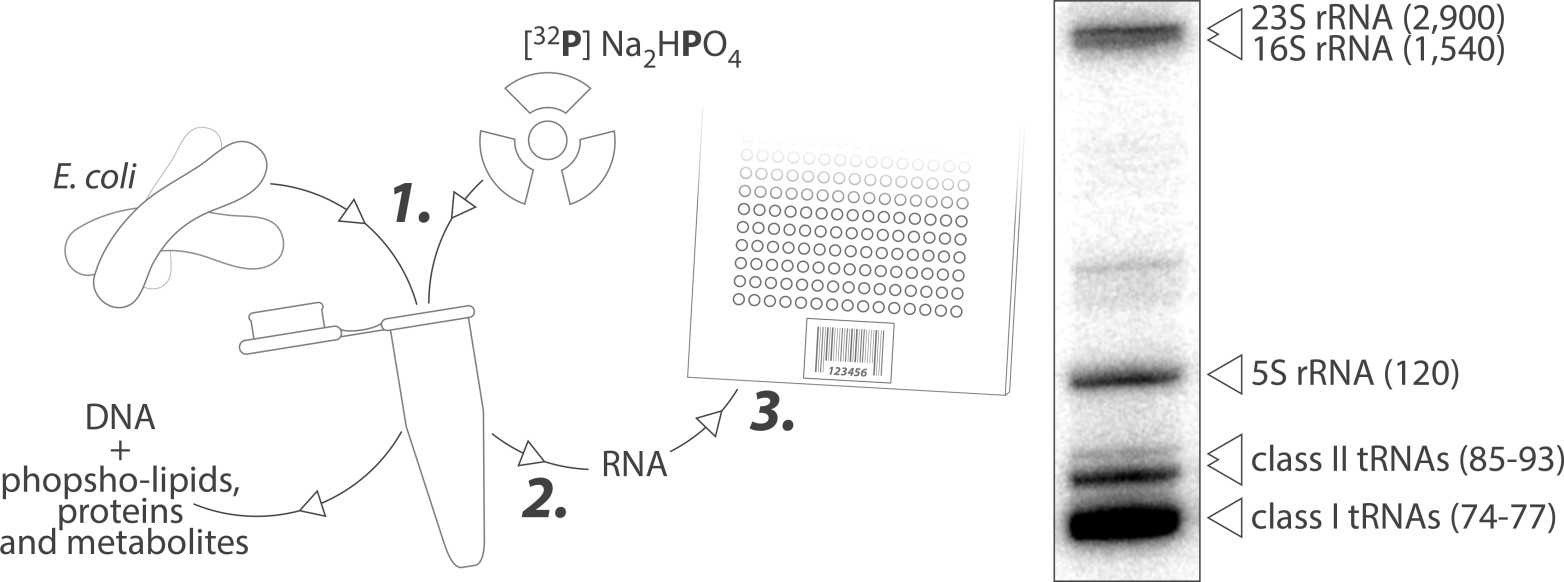
Schematic description of the three-step method used to profile tRNA expression in *E*. *coli*. (1) *E*. *coli* cultures were spiked with radioactive orthophosphate and grown to OD_600nm_ = 0.5. (2) Total RNAs are extracted and separated from other biomolecules with Trizol reagent. (3) Total RNAs were separated on 10% denaturing PAGE or hybridized on custom tRNA microarray and quantified by phosphorimaging. The molecular ratio tRNA to 5S rRNA (large ribosomal subunit) is 15 to 1. The low apparent ratio 16S (small subunit) and 23S (large subunit) to 5S results from a frequently observed bias of the Trizol extraction against high molecular weight RNAs.

*E*. *coli* was grown under aerobic conditions in LB media supplemented with 1 to 20 μCi of [^32^P] Na_2_HPO_4_ and harvested at OD_600nm_ = 0.5 (exponential phase). Total RNAs were purified by Trizol extraction and loaded on 10% denaturing PAGE or hybridized on tRNA microarrays (Fig 1). Radioactive material in the tested range was incorporated at approximately 25% in whole-cell extracts and 1% in purified RNAs as measured by scintillation counting (S2 File).

**Fig 2:**
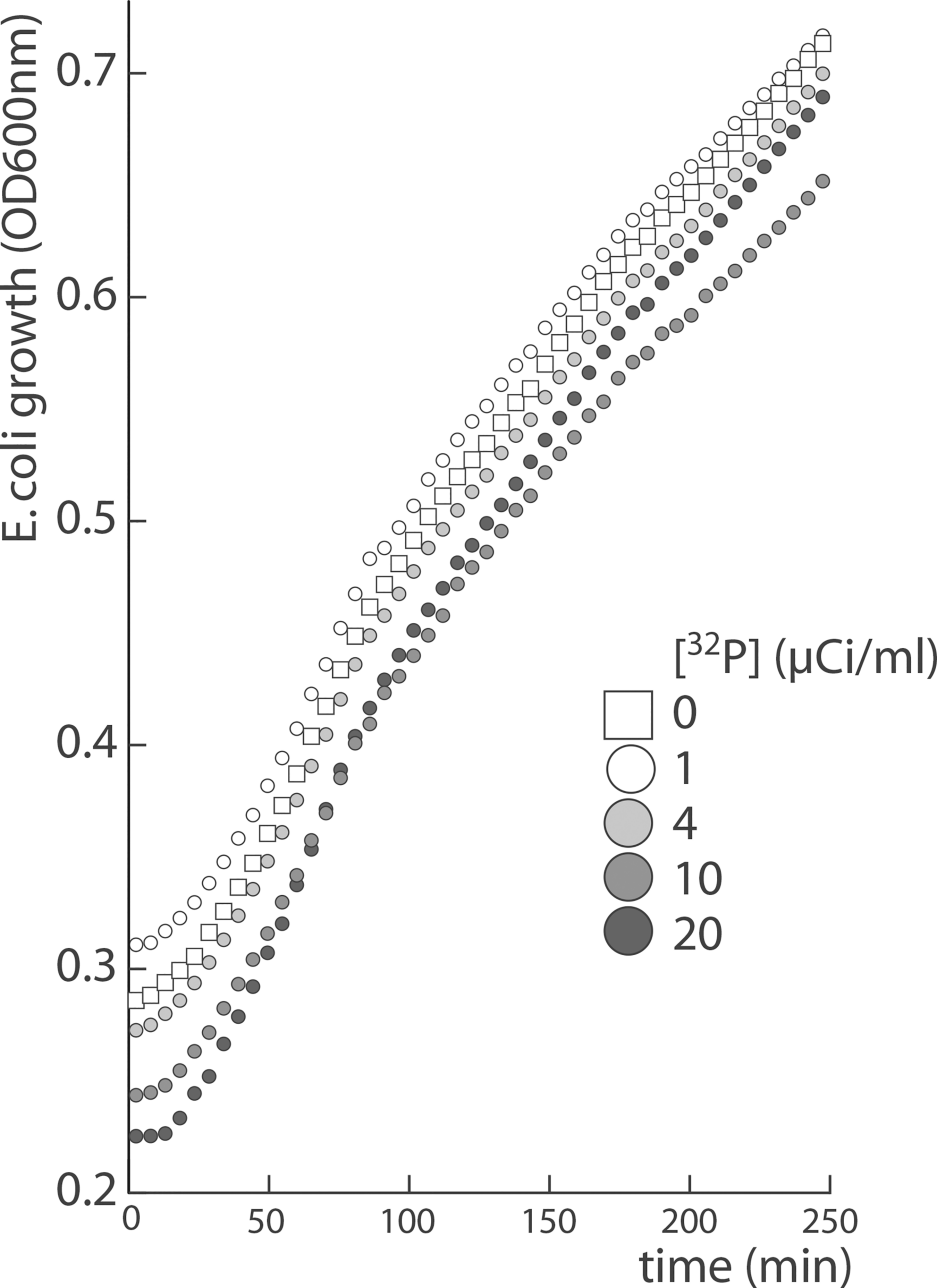
*In vivo* radiolabeling is innocuous toward bacterial growth. 250 μl of *E*. *coli* cultures spiked with 1 to 20 μCi/ml of [^32^P] Na_2_HPO_4_ were grown in triplicate in 96-well plates. Optical density was monitored at 600nm using a multi-well incubator-reader. Radiations in the tested range have no significant effect on growth rate.

Ionizing radiation such as beta particle emissions are known bacteriostatic and bactericidal agents [20]. *E*. *coli* growth rate was monitored in 96-well plates to investigate whether the presence of radioactive orthophosphate could potentially generate suboptimal growth conditions and therefore bias tRNA profiles. Radiolabeling conditions in the tested range were found to be innocuous to bacterial cells (Fig 2). The amount of radioactive material for metabolic labeling was set at 10 μCi/ml and used in all subsequent experiments.

*In vivo*, all four ribonucleotides are synthesized *de novo* or recycled through salvage pathways [21]. *De novo* synthesis relies on ribose 5-phosphate, a product of the pentose phosphate pathway. This phosphorylated ribosyl-unit constitutes the main incorporation route of ^32^P into nucleic acids. Because all cellular RNAs are assembled indiscriminately from neosynthesized, or recycled building blocks, they incorporate radioactive nucleotides at the same rate. For RNAs of equivalent sizes such as tRNAs, abundance of individual species is directly proportional to their radioactive activity. LB is a complex media and by definition its exact chemical composition is unknown. The closest defined media is M9 containing 22 mM of KH_2_PO_4_. By extrapolation, addition of 10 μCi/ml final in LB, represents a 2,000-fold isotopic dilution of stock [^32^P] Na_2_HPO_4_ (1 μCi/μl, 1 Ci/mmol). Under such conditions, tRNA molecules statistically carry a maximum of one radioactive phosphorus atom per molecule.

### Reproducibility and specificity

**Fig 3:**
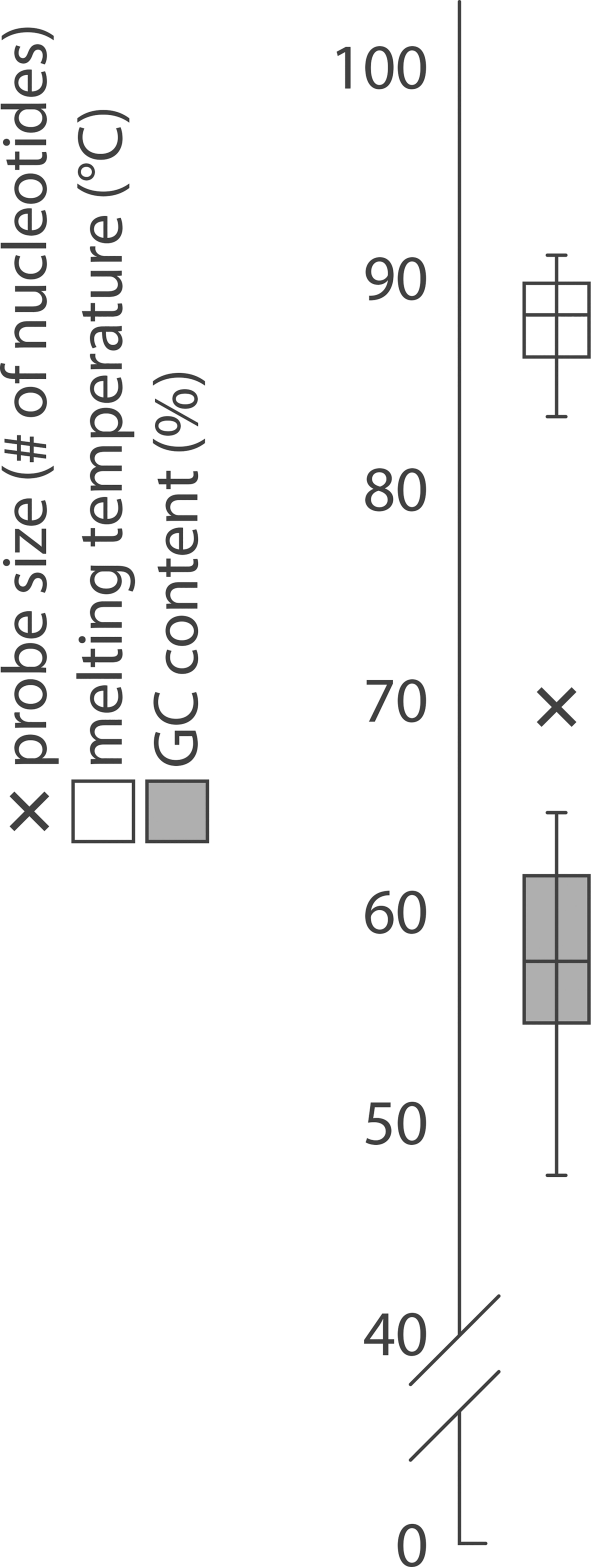
Physicochemical properties of microarray DNA probes. All 42 DNA probes are 70 nucleotides long and complementary to the 3’ end of the different *E*. *coli* tRNA (3’ CCA excluded). Their calculated average melting temperature is 87 +/- 2°C. Their average GC content is 57 +/- 4%. Uniform probe features are key to equivalent hybridization capacity and direct measurement of tRNA abundance from spots signal.

It has been shown that 70-mer DNA probes complementary to the tRNA 3’ end (CCA excluded) are sufficient to distinguish species that differ by about 8 or more residues [15]. Conveniently, the majority of *E*. *coli* tRNAs display great variation in their primary structures which simplified the design of DNA probes and minimized undesirable tRNA cross-hybridizations. Forty-two probes were sufficient to capture the 48 *E*. *coli* tRNA sequences on the arrays. Six probes specific to tRNA Leu (CAG), Met initiator, Met elongator, Tyr (GTA), Val (GAC) and Thr (GGT) were degenerated at one to five positions in order to hybridize all corresponding isodecoders—tRNA sharing identical anticodons but displaying slight variations in their body sequence (See S1 File for probe sequences). All probes shared an identical size and comparable physicochemical properties such as melting temperatures and GC content (Fig 3). In addition, they were spotted, hybridized and washed using uniform protocols. Under such conditions, each probe had equivalent hybridization ability supporting the unbiased measurement of tRNA levels directly from spot signals.

**Fig 4:**
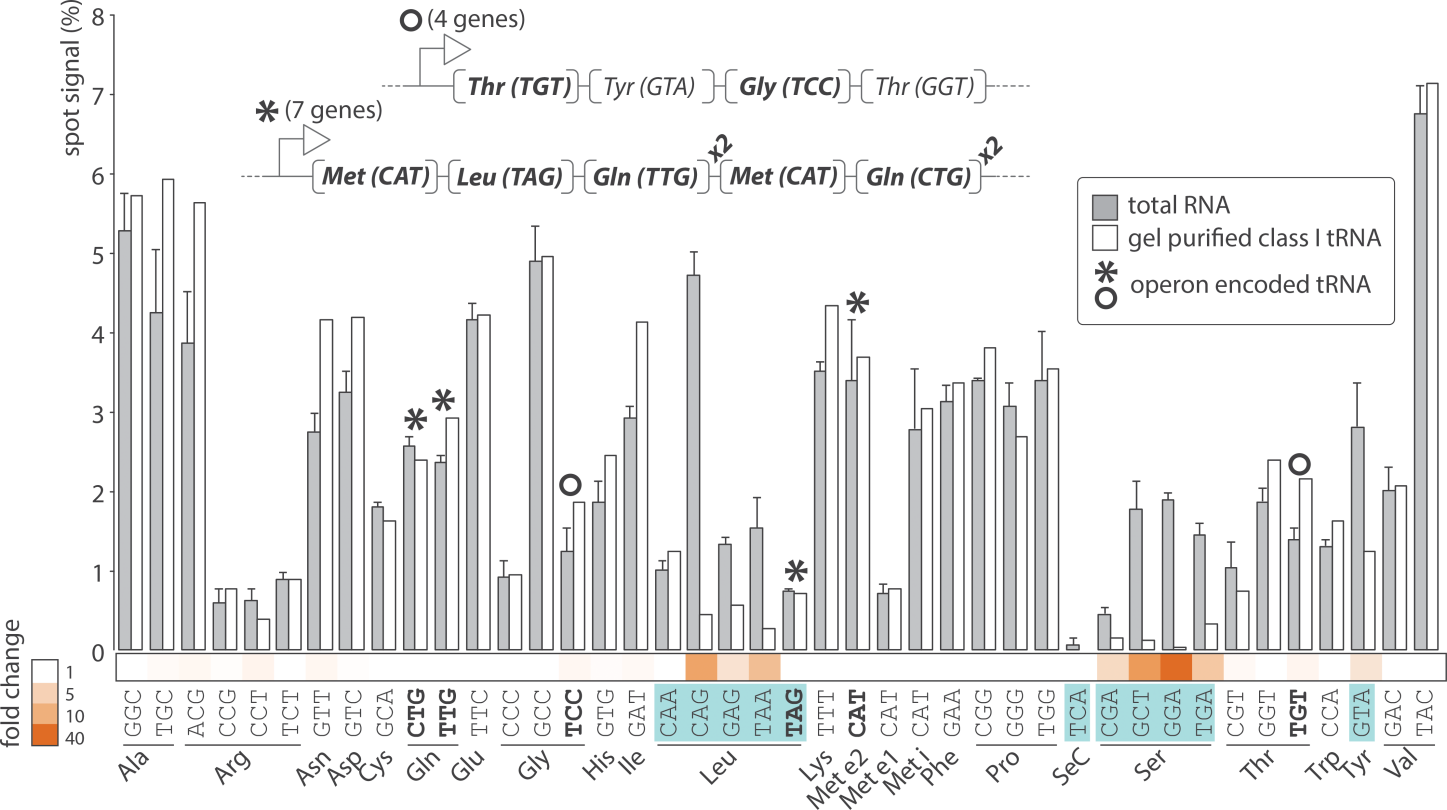
Relative abundance of 42 tRNA species in *E*. *coli*. The bar graph reports spot signal for each probe, which is proportional to tRNA abundance, in total RNA (grey) and gel purified class I tRNA (white). Spot signal for each probe is represented as a fraction of all signals combined expressed in %. tRNA abundance in total RNA sample is the average of three independent biological replicates (corresponding standard deviation are indicated). Shades of orange illustrate the magnitude of the drop in tRNA abundance between total and gel purified samples. The top panel describes the topography of two operons (marked with ° and *) encoding exclusively tRNA genes. Genes in bold have no other copy outside these two transcriptional units. Class II tRNAs are highlighted in blue.

**Fig 5:**
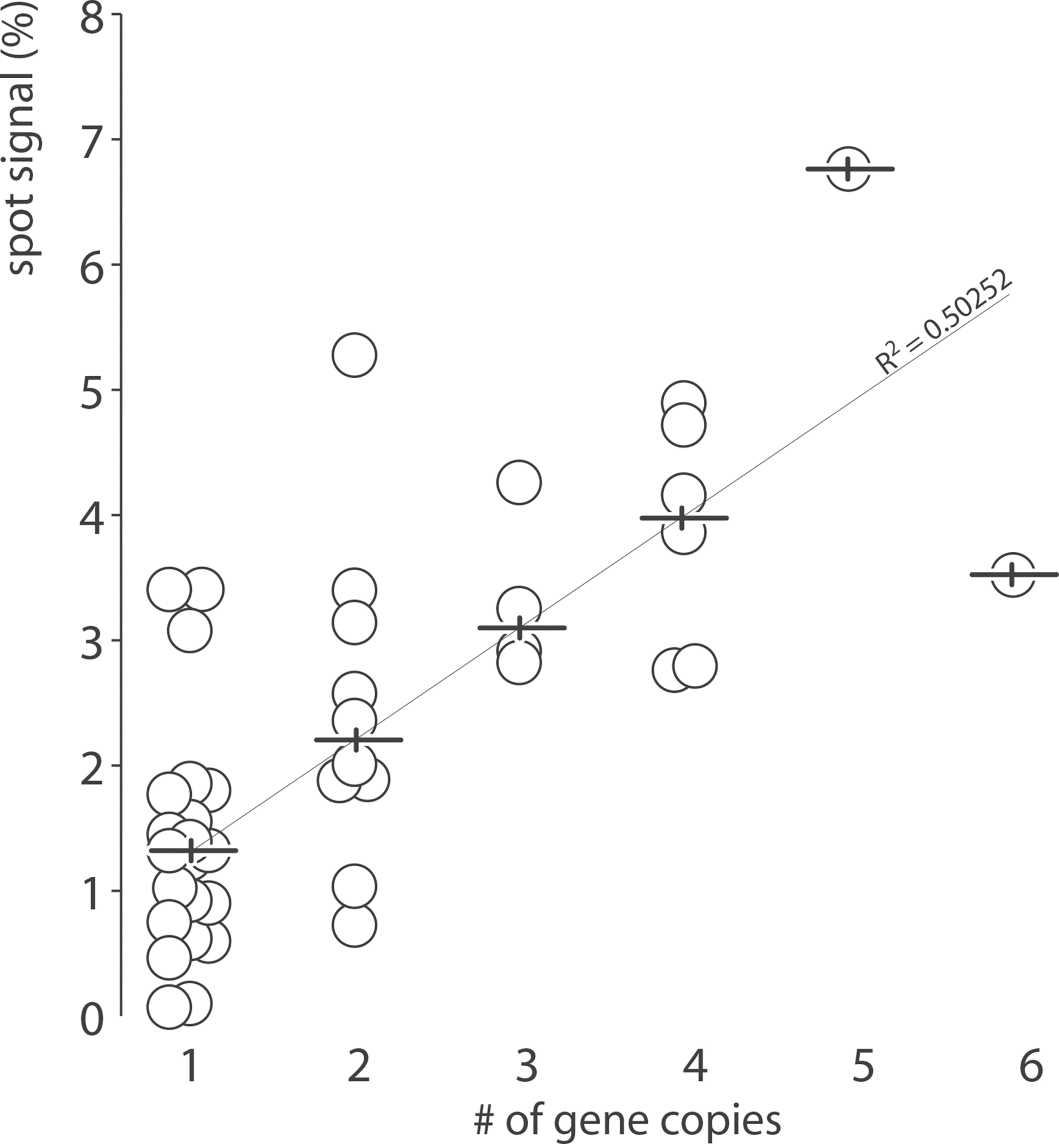
tRNA abundance versus tRNA gene copy number. *E*. *coli* tRNA genes are organized in 6 different groups according to their number of copies. Horizontal bars indicate the median tRNA abundance (from Fig 4) for each group.

Arrays prepared manually consisted in eight replicates per probe for a total of 336 spots. Signal intensity for each metabolically labeled tRNA was measured by phosphorimaging and normalized to the signal of all spots combined for straightforward array-to-array comparison. Under optimal growth conditions, all tRNAs were significantly expressed above detection threshold (Fig 4, grey bars) with the exception of tRNA selenocysteine (SeC (TCA)) which barely surpass background levels, an observation consistent with published data [22]. SPOt showed a wide range of probe signals with a 15-fold difference between the highest (Val (TAC)) and the lowest abundant tRNA (Ser (CGA)). Expression within isoacceptor families varied greatly. For example, all three Proline isoacceptors were expressed at a comparable level, whereas a 6-fold difference was observed between the highest and lowest expressed Arginine and Leucine accepting species. tRNA levels increased with gene dosage, displaying a notable dispersion for single and double gene copies as reported in published studies [23] (Fig 5 and S3 File).

Five mixed tRNA/rRNA operons encode fourteen tRNA genes transcribed as six different tRNA species [24]. The average level of tRNA co-transcribed with rRNA is 1.13% ± 0.17 per gene copy as opposed to 1.29% ± 0.78 for tRNA genes outside these transcriptional units, suggesting that polycistronic arrangements with other elements of the translation machinery favors steady tRNA expression. *E*. *coli* genome contains also several operons encoding tRNA genes exclusively [25]. Two of them are of particular interest as they cluster all the copies of a given tRNA gene. They offer the opportunity to investigate the correlation of gene copy number and tRNA expression without the bias of chromosomal positioning. The operon Thr (TGT)–Tyr (GTA)–Gly (TCC)–Thr (GGT) encodes the only copy of tRNA Thr (TGT) and Gly (TCC) (Fig 4, upper panel). These two tRNAs were expressed at equivalent level with 1.39 and 1.25% respectively. Similarly, the operon Met (CAT)–Leu (TAG)–Gln (TTG)x2 –Met (CAT)–Gln (CTG)x2 encodes the only copies of the four corresponding tRNA genes (Fig 4, upper panel). As predicted, glutamine and methionine tRNA encoded by two gene copies were expressed at a significantly higher level as compared to Leucine tRNA encoded by a single copy gene.

Array analysis of three independent biological replicates revealed a median probe signal of 2.01% and an associated median standard deviation of 0.18%. The observed false changes due to inherent technical errors were comprised between 1% (Pro (CGG)) and 32% (Thr (CGT)) with a median at 9%. These values were substantially lower than reported changes in tRNA expression interpreted as biologically significant in recent publications [8, 17]. Nevertheless, SPOt was subjected to further quality assessments. First, proportions of class I and class II tRNA species were estimated as a whole on denaturing PAGE and compared to the corresponding combined probe signals on the arrays (Table 1). The two independent techniques revealed nearly identical tRNA distributions. Second, class I tRNAs were gel purified and hybridized on arrays independently from other cellular RNAs. As expected, levels of class II tRNAs dropped by 2 to 37-fold and class I tRNAs raised accordingly by 1.1-fold (Fig 4, white bars). Interestingly, levels of tRNA Leu (CAA) and (TAG) remained steady. With only 85 nucleotides, these two tRNAs are at least two nucleotides shorter than any other class II species, suggesting that they could have been inadvertently co-extracted along with class I tRNAs and hybridized on the array. Finally, removing abundant ribosomal RNA had no effect on the signal to noise ratio further justifying the streamlined hybridization of total RNA samples.

**Table 1:** Proportion of class I and class II tRNA measured by gel electrophoresis and microarrays.

	class I tRNA (%)	class II tRNA (%)
denaturing PAGE	81.4 ± 0.5	18.6 ± 0.5
array	82.1 ± 1.5	17.8 ± 1.5

The table indicates median and standard deviation of three independent biological replicates.

### Dynamic range and sensitivity

**Fig 6:**
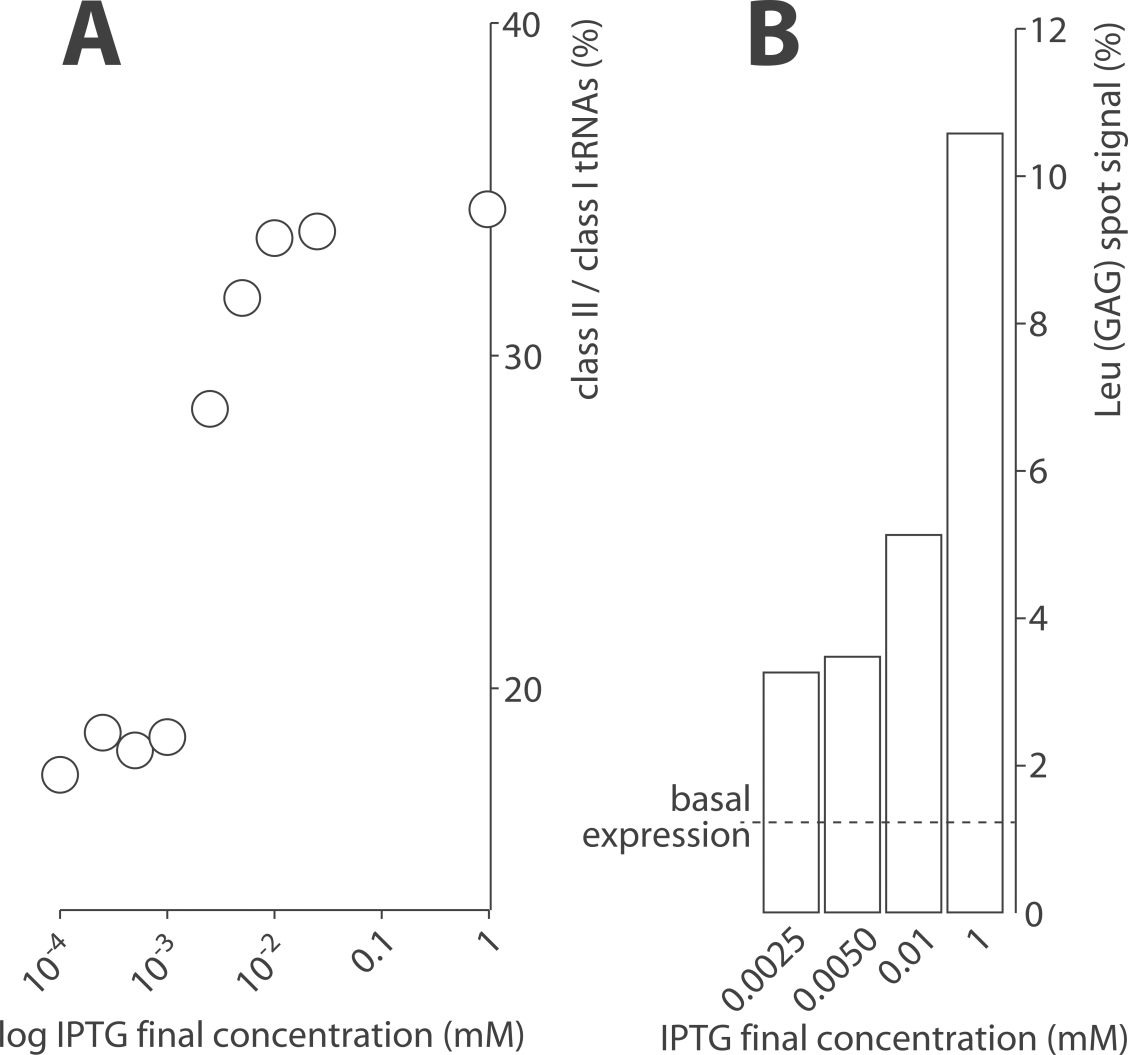
Controlled tRNA Leu (GAG) overexpression monitored by gel electrophoresis and microarray. (A) Nine different IPTG concentrations were tested in order to establish the boundaries of the dose response. Total RNAs were extracted from induced recombinant *E*. *coli* cultures. Class I and class II tRNAs were separated on 10% denaturing PAGE, stained with toluidine blue and quantified by gel imaging. The ratio class II over class I tRNA was used as a proxy for tRNA Leu (GAG) overexpression. (B) The bar graph indicates the level of tRNA Leu (GAG) as measured by microarray in recombinant cells induced with IPTG concentrations ranging from 2.5 μM to 1 mM. The dashed line indicates the basal expression level in absence of IPTG.

*E*. *coli* tRNA Leu (GAG) was over-expressed *in vivo* from an inducible pTrc99 plasmid using IPTG concentrations ranging from 0.1 μM to 1 mM in non-radioactive cultures. The corresponding increments in class II tRNAs were monitored by gel electrophoresis and gel imaging of RNA bands stained with toluidine blue. Proportion of class II tRNAs increased from 2.5 μM IPTG on and eventually plateaued in the high micromolar range (Fig 6A). Overexpression had no apparent incidence on cell growth rate. An equivalent dose response was observed with microarrays in cultures supplemented with [^32^P] Na_2_HPO_4_ (Fig 6B). Levels of tRNA Leu (GAG) gradually increased up to 8-fold compared to basal expression providing further validation of our experimental setup.

**Fig 7:**
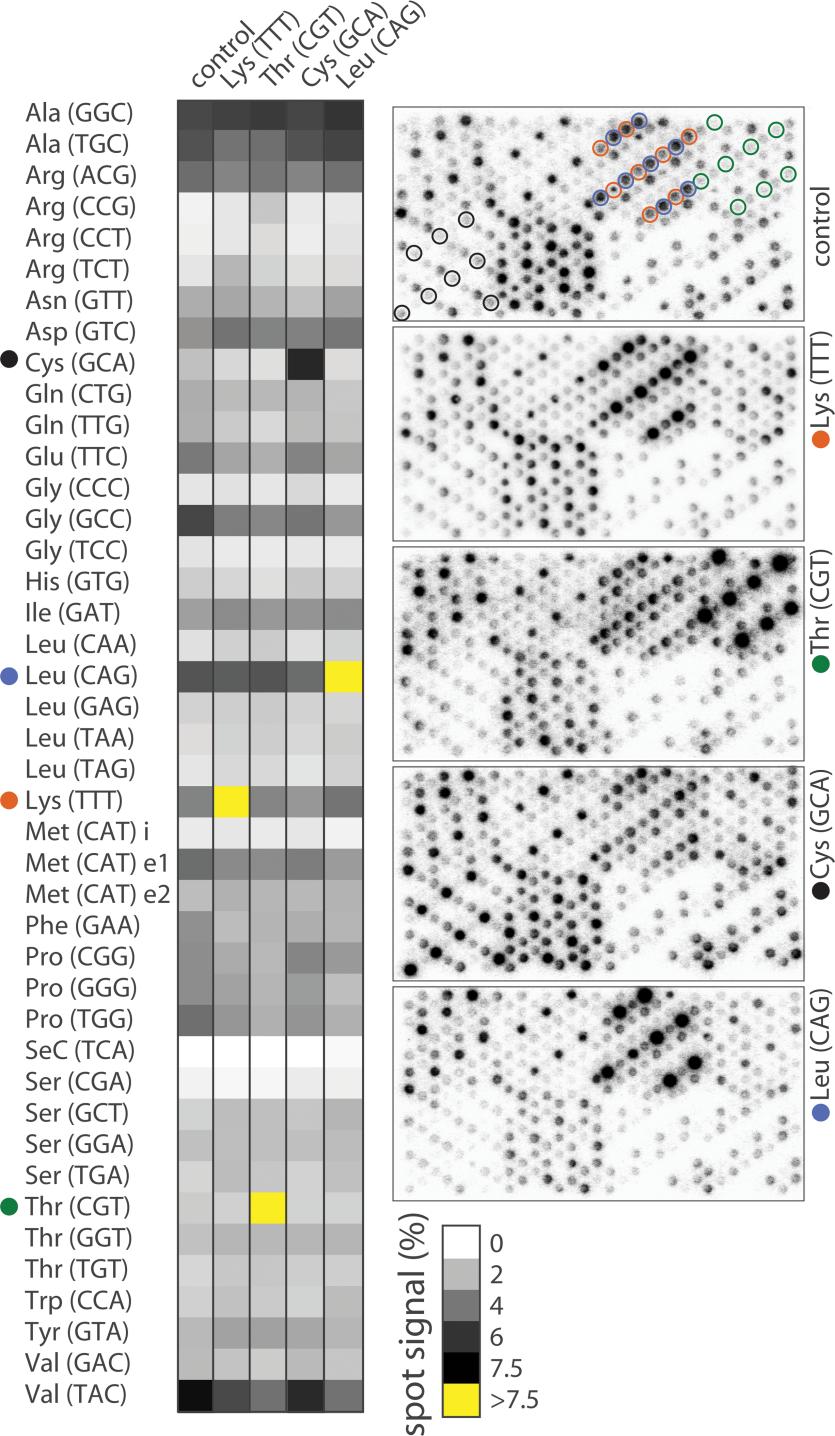
tRNA profiles in tRNA overexpressing cells. *E*. *coli* was transformed independently with four plasmids encoding *E*. *coli* Lys (TTT), Thr (CGT), Cys (GCA) or Leu (CAG) tRNA genes. Transgene expression was turned on with 0.5 mM IPTG and synchronized with metabolic labeling. tRNA levels are represented as shades of grey on the heat map. Yellow indicates levels higher than 7.5% (the highest level for an individual tRNA in the control sample). Arrays for control and overexpressing cells are displayed on the right. Spots (8 per probe) specific to Lys (red), Thr (green), Cys (black) and Leu (blue) tRNA are circled on the control array (top array).

Four additional *E*. *coli* tRNA, namely Leu (CAG), Lys (TTT), Cys (GCA) and Thr (CGT), were expressed from pTrc99 plasmids using a fixed and non-limiting IPTG concentration. Overexpression did not significantly affect the signal of non-specific probes especially the ones hybridizing isoacceptors, which are closer in sequence and therefore particularly sensitive to cross-hybridization (Fig 7). Three of these constructs supported tRNA expression at levels beyond any endogenous species (Table 2) without impacting surrounding probes, further validating the method of background subtraction. In particular, abundance of artificially expressed tRNA Leu (CAG) was two-fold above endogenous tRNA Val (TAC), the highest expressed tRNA, placing the saturation threshold comfortably above any array data points collected under physiological conditions.

**Table 2:** Characteristics of overexpressed tRNAs.

	level (%)	overexpression (fold)	gene copies
Leu (CAG)	12.6	2.6	4
Lys (TTT)	9.1	2.7	6
Cys (GCA)	6.3	5.0	1
Thr (CGT)	11.6	8.4	2

The table indicates overall tRNA levels (combined expression from plasmid and chromosome), fold change in expression compared to control and number of chromosomal gene copies.

**Fig 8:**
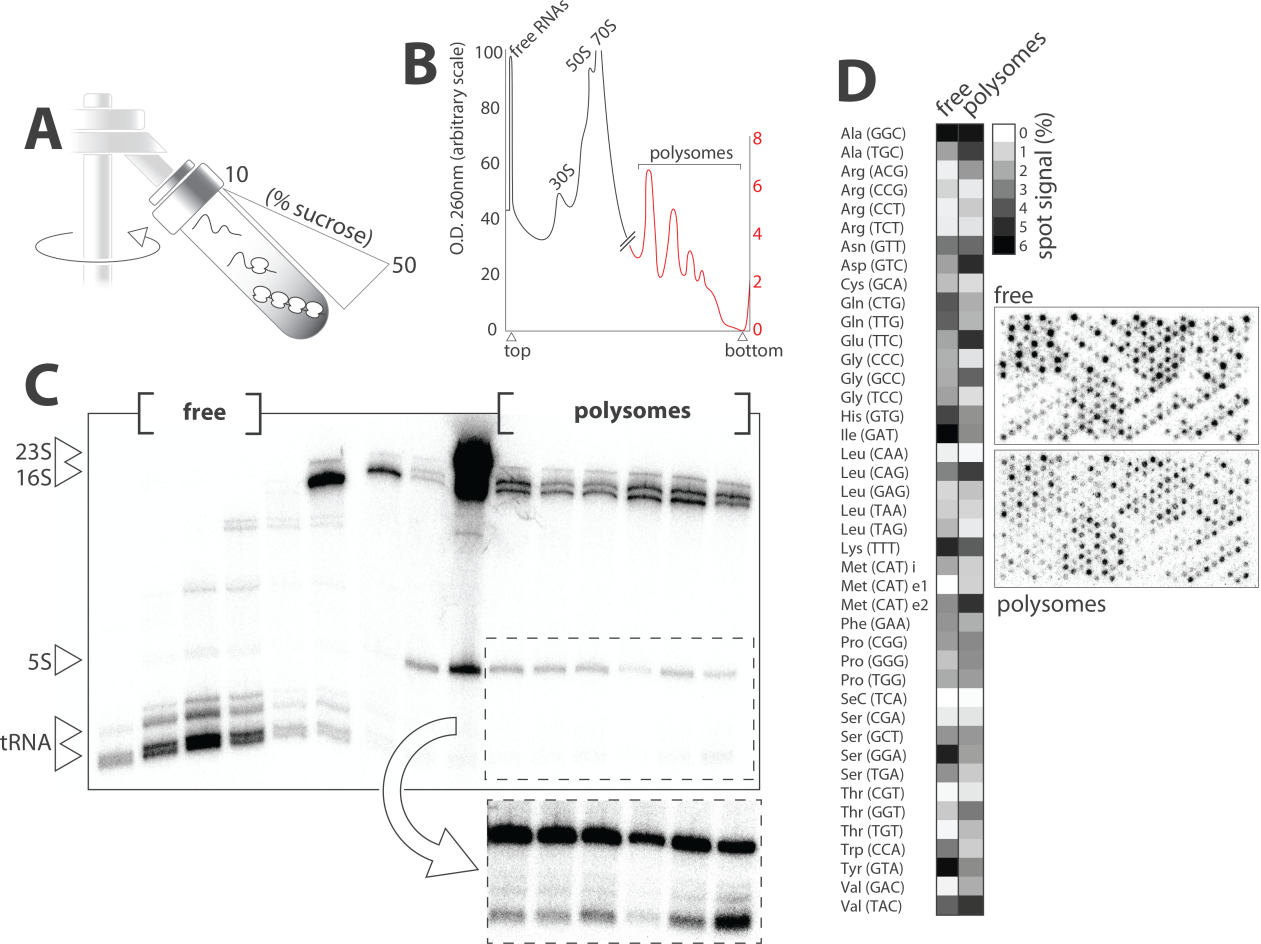
tRNA profiles in polysomes. (A) Lysates from *E*. *coli* grown in presence of [^32^P] Na_2_HPO_4_ were loaded on sucrose gradient and centrifuged. (B) Nucleic acids content along the gradient was continuously analyzed at 260 nm (the corresponding graph is a vectorized copy of a printed-paper chart). Note the two different scales: black for low molecular weight fractions and red for polysomal fractions. (C) Recovered fractions were separated on 10% denaturing PAGE and visualized by phosphorimaging. Longer exposure was necessary to observe polysome-associated tRNAs on the gel (dashed box). (D) Fractions corresponding to free and polysomal tRNA were pooled and hybridized separately on microarray. The heat map shows the abundance of the different *E*. *coli* tRNA in both samples. Top array (free) was exposed overnight as opposed to four days for bottom array (polysomes) due to low signal.

**Fig 9:**
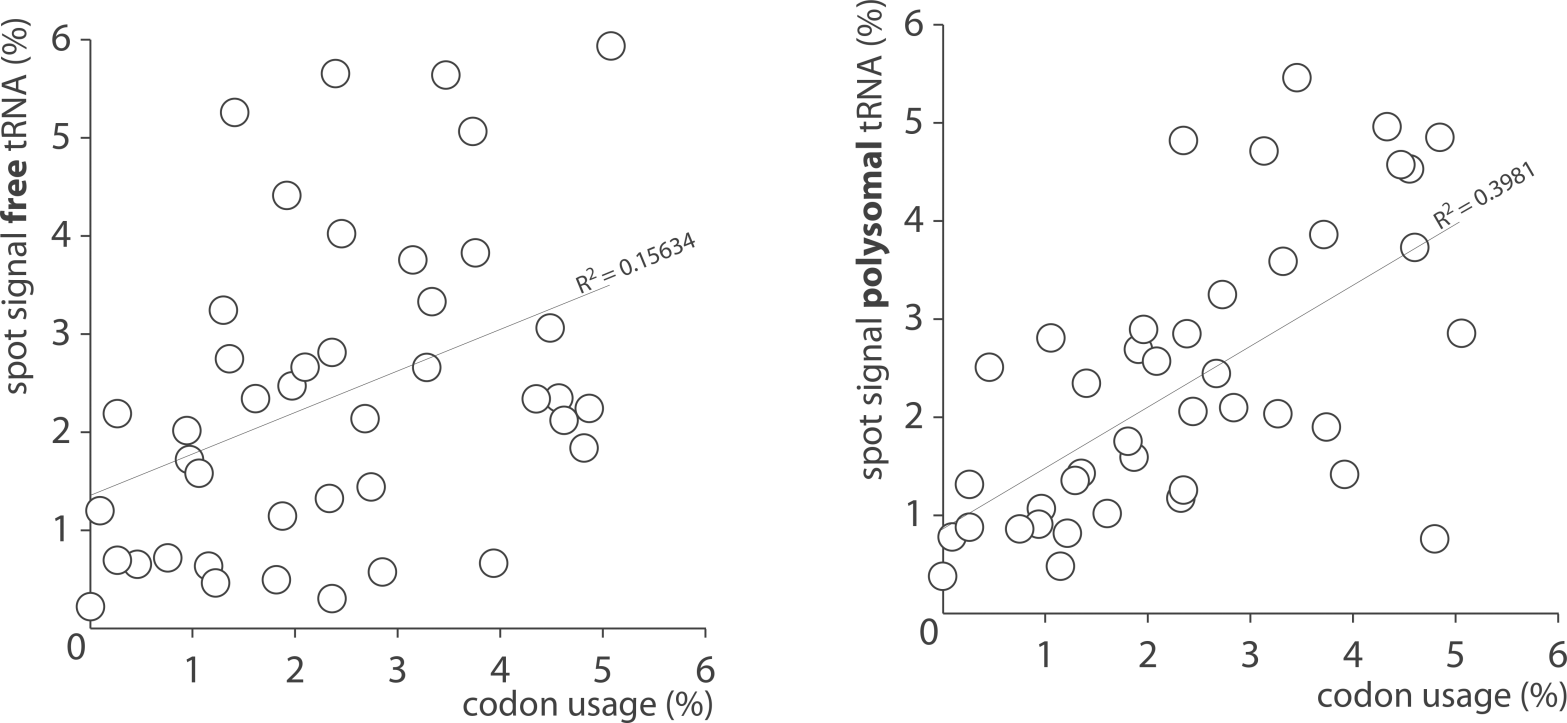
tRNA abundance versus frequency of codon usage. tRNA abundance in free and polysomal fractions are from Fig 8D. Frequency of codon usage was adapted from the Genomic tRNA Database to take into account both Watson-Crick and wobble base-pairing in codon-anticodon interactions. Our data suggest that translating ribosomes operate some form of tRNA discrimination in order to optimize codon translation.

It has been shown in multiple species that the subpopulation of tRNAs physically interacting with ribosomes and therefore actively engaged in translation represent only a small minority of the total tRNA pool. In *E*. *coli* grown under optimal conditions, the molar ratio tRNAs to 5S rRNAs was approximately 15:1 as estimated by gel electrophoresis of metabolically labeled RNAs (Fig 1). Polysomal fractionations were performed to test whether the expected low signals of ribosome-associated tRNAs were an obstacle for microarray profiling (Fig 8A). Metabolically labeled bacterial lysates were loaded onto sucrose gradient, centrifuged and fractioned as detailed in the material and methods section. The distribution of nucleic acids along the gradient was monitored continuously at 260 nm (Fig 8B). Fifteen fractions were subjected to Trizol-extraction, and total RNA were separated on denaturing PAGE and analyzed by phosphorimaging (Fig 8C). The method allowed isolating samples containing exclusively free tRNAs (or engaged in low molecular complexes), small (30S) and large (50S) ribosomal subunits, monosomes (70S) or polysomes. Samples containing free and polysomal tRNAs were then hybridized separately on microarrays and analyzed by phosphorimaging (Fig 8D). The corresponding profiles were significantly different suggesting that translating ribosomes operated some form of tRNA selection. The biological relevance of this observation was further investigated by plotting measured tRNA expression levels with predicted codon usage (Fig 9). Codon frequency correlated poorly with levels of free tRNAs compared to polysomal tRNAs (R^2^ = 0.15 and 0.39 respectively) supporting the idea that translating ribosomes selectively siphon a subpopulation of tRNA from the total tRNA pool in order to optimize mRNA translation.

## Conclusion

SPOt is a novel and cost effective approach that allows measuring tRNA levels in only three steps and in less than 24 hours. The extensive testing performed in this study showed that SPOt has a large dynamic range, is highly reproducible and specific. Because the detection threshold is easily adjusted by modulating array exposure times, SPOt is also perfectly adapted to profile low abundant species such as tRNAs associated with polysomes.

Current platforms for tRNA profiling measure relative tRNA expression or the change in tRNA levels between a sample of interest and a predefined reference. Fold changes in tRNA expression are calculated and interpreted uniformly across the tRNA pool without consideration of absolute amounts. For example, a low and a highly abundant tRNA could display identical fold changes between two samples whereas the difference in the number of tRNA molecules is in reality vastly different. In addition, detection of low abundant tRNAs is inherently prone to technical errors which further biases interpretation [23]. SPOt measures absolute amounts of tRNA. Low abundant species are identified and the biological relevance of changes in their expression can be objectively appreciated.

**Fig 10:**
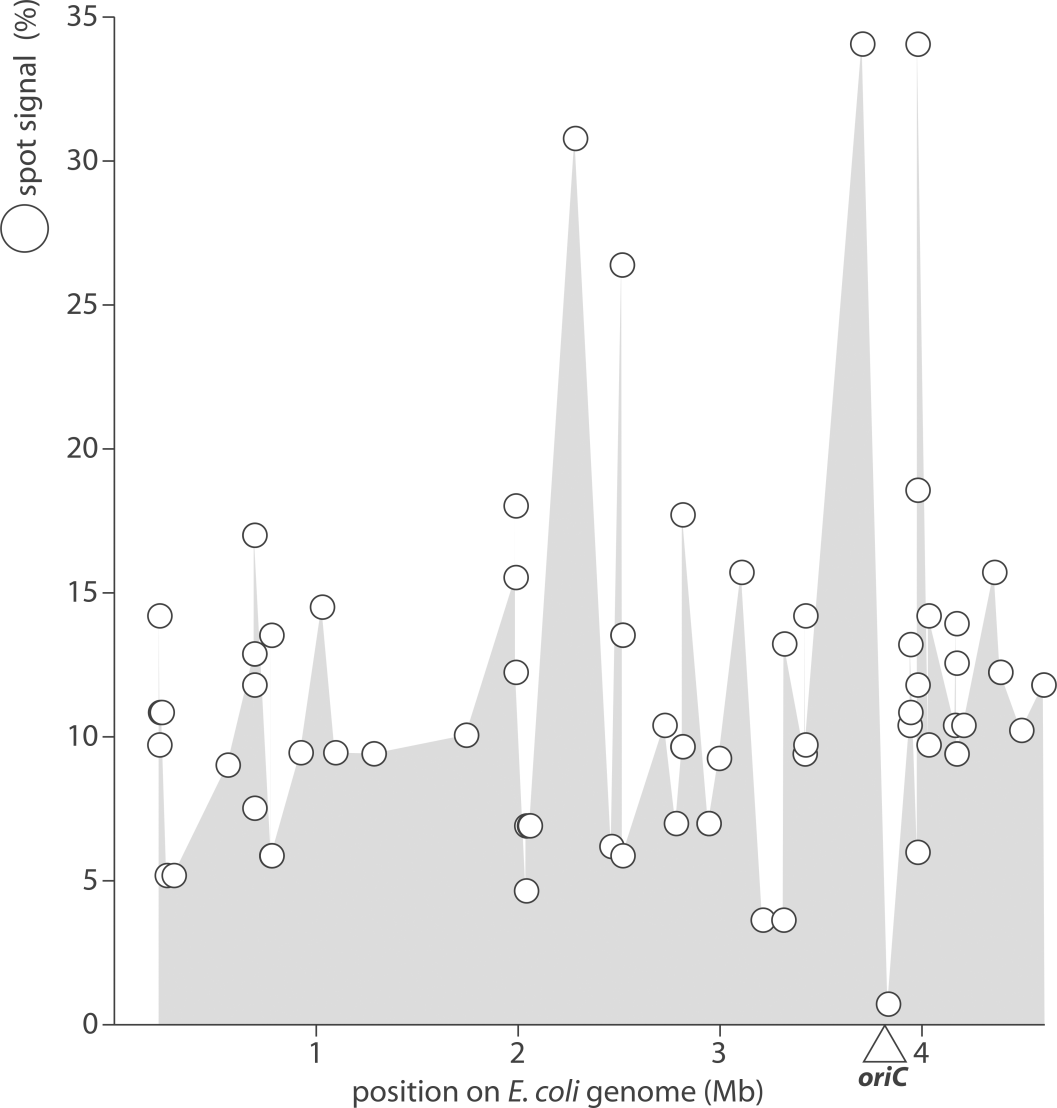
tRNA abundance versus chromosomal position of tRNA genes. tRNA levels (white circles) are indicated on the right y-axis. Levels from Fig 5 were normalized to the number of corresponding gene copies. tRNA gene loci are from the Genomic tRNA Database. The position of the origin of replication (oriC) is indicated.

SPOt also informs indirectly on the transcriptional activity of 86 loci spanning the entire *E*. *coli* chromosome (Fig 10). tRNA genes clustered around the origin of replication show a high variation in expression that could be attributed to targeted transcription regulation or general modulation of chromatin structure.

Finally, SPOt is a versatile platform that can be adapted to any kind of organisms as long as a genome is available for probe design. Species that are grown routinely in laboratory settings would represent ideal candidates for metabolic labeling. The relevance of this tool to profile tRNAs in cultured human and mouse cells is under investigation. The amount of RNAs extracted from adherent mammalian cells is typically lower compared to organisms grown in suspension such as *E*. *coli*, so cultures need to be substantially scaled up. In addition, the tRNA landscape is more complex in mammals which genomes encode many more isodecoders (tRNAs that share identical anticodons but display small differences in their body sequences). Consequently probes are partially degenerated to allow homogenous hybridization of tRNA species that differ by less than eight nucleotides. Sequences for human and mouse microarray probes are published and available to the scientific community.

## Supporting information

S1 File Probe sequences and microarray layout. (XLS)

S2 File Efficiency of ^32^P incorporation in RNAs. (TIF)

S3 File Processed tRNA microarray data. (XLSX)
