# Supplementary figures and images for: SPOt: A novel and streamlined microarray platform for observing cellular tRNA levels
Source: PLoS One. 2017 May 17;12(5):e0177939. doi: 10.1371/journal.pone.0177939 (PMC5435355; doi:10.1371/journal.pone.0177939)

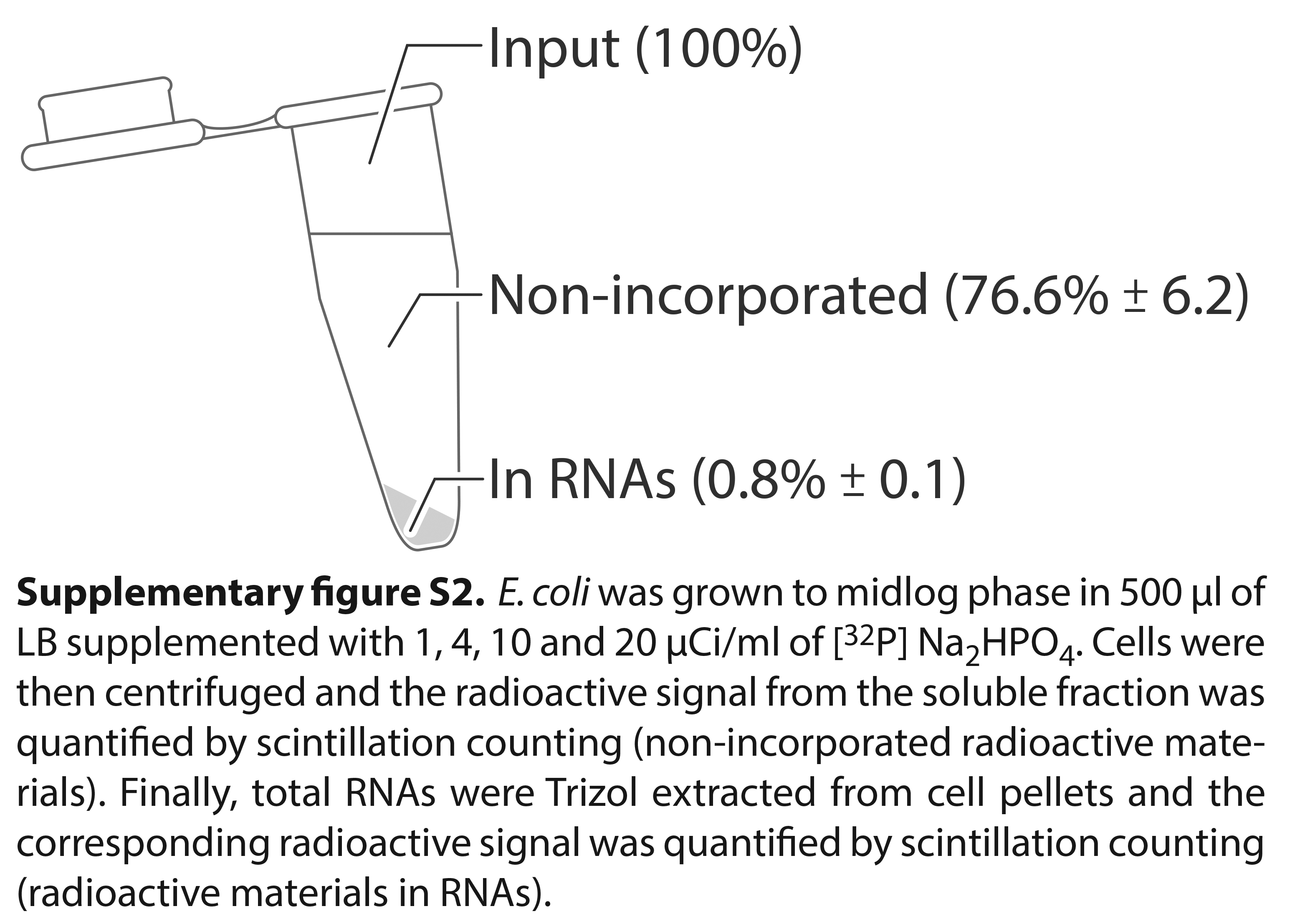

Supplement: S2 File — (TIF) [file pone.0177939.s002.tif]
